# Minimizing catecholamines and optimizing perfusion

**DOI:** 10.1186/s13054-019-2433-6

**Published:** 2019-06-14

**Authors:** Daniel De Backer, Pierre Foulon

**Affiliations:** Department of Intensive Care, CHIREC Hospitals, Université Libre de Bruxelles, Boulevard du Triomphe 201, B-1160 Brussels, Belgium

**Keywords:** Cardiac output, Microcirculation, Vasopressor agents, Inotropic agents, Fluid therapy

## Abstract

Catecholamines are used to increase cardiac output and blood pressure, aiming ultimately at restoring/improving tissue perfusion. While intuitive in its concept, this approach nevertheless implies to be effective that regional organ perfusion would increase in parallel to cardiac output or perfusion pressure and that the catecholamine does not have negative effects on the microcirculation. Inotropic agents may be considered in some conditions, but it requires prior optimization of cardiac preload. Alternative approaches would be either to minimize exposure to vasopressors, tolerating hypotension and trying to prioritize perfusion but this may be valid as long as perfusion of the organ is preserved, or to combine moderate doses of vasopressors to vasodilatory agents, especially if these are predominantly acting on the microcirculation. In this review, we will discuss the pros and cons of the use of catecholamines and alternative agents for improving tissue perfusion in septic shock.

## Introduction

Shock, whatever its cause, is characterized by a decrease in tissue perfusion, associated with cellular and metabolic abnormalities. The base of shock resuscitation is to improve tissue perfusion by restoring perfusion pressure of vital organs, ensuring an adequate cardiac output and, if possible, improving microvascular alterations. Several interventions can be considered, including fluids, vasopressor, and inotropic agents. Each of these interventions has adverse effects. A positive fluid balance is associated with an increased risk of death [1]. Interestingly, this study reported that the positive fluid balance was mostly due to a lower fluid output (mostly diuresis) while the amount of fluids administered to the patients did not differ between survivors and non-survivors. Hence, it is impossible to differentiate the impact of disease severity from a direct negative effect of fluids. Nevertheless, it sounds reasonable to avoid excessive fluid administration. As an alternative to fluids, vasoactive agents can be proposed. Vasopressive and inotropic catecholamines are life-saving interventions in several circumstances, but these agents are also associated with important adverse events. Tachycardia, arrhythmias, and metabolic, thermogenic, and immunologic effects are the most noticeable ones, but other adverse effects may also occur. In addition, vasopressive catecholamines may be associated with excessive vasoconstriction which may result in an impairment in tissue perfusion, even when perfusion pressure is restored. However, non-adrenergic vasopressors are also associated with some adverse effects, and switching from adrenergic to non-adrenergic vasopressors is not always optimal. Similarly, non-catecholaminergic inotropic agents may also be associated with adverse effects including tachycardia, digital necrosis, or splanchnic ischemia. Accordingly, the challenge in managing critically ill patients is to find the right balance between fluids, adrenergic and, eventually, non-adrenergic vasopressors, and inotropic agents. In this paper, we will focus on septic shock and discuss the different possibilities to optimize tissue perfusion and catecholamine use.

## Manipulating perfusion pressure

Several trials have demonstrated that the severity and duration of hypotension are associated with a poor outcome [2, 3]. It sounds reasonable to introduce vasopressors without delay as the late introduction of vasopressors seems to be associated with an increased risk of death [4].

**Fig. 1:**
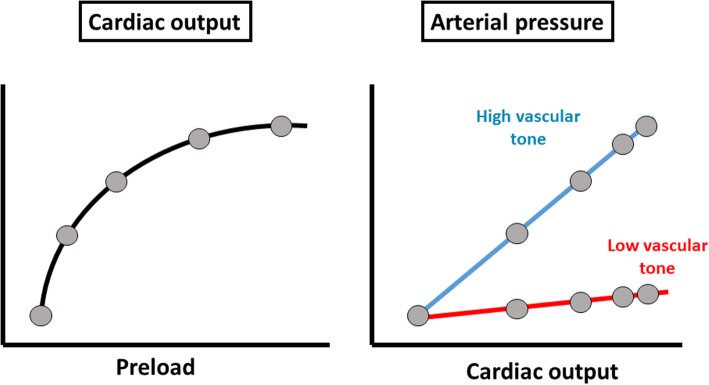
Effects of fluids on arterial pressure. Fluids increase preload which in turn can increase cardiac output if the heart operates on the steep part of the Starling relationship. Whatever the increase in cardiac output, fluids increase arterial pressure mostly in patients with high vascular tone. In patients with low vascular tone, even major increases in cardiac output have minimal impact on arterial pressure

Fluids can increase perfusion pressure, but this effect is rather indirect, as it is mediated by an increase in cardiac output and requires that vascular tone is not too low (Fig. 1). Measurements of arterial elastance (defined as arterial pressure variations divided by stroke volume variations over one respiratory cycle) have been demonstrated to predict the pressure response to fluids [5], but these measurements are not easy to perform in clinical practice. Ideally, a beat-by-beat measurement of cardiac output independent of the arterial waveform should be obtained to prevent mathematical coupling of data using arterial trace to derive pulse pressure and stroke volume. Due to these methodological limitations, this principle is not used at bedside routinely and, in practice, most physicians perform fluid challenges evaluating whether or not perfusion pressure would increase with fluid administration. One may also use diastolic pressure as a witness of the alteration in vascular tone. When diastolic pressure is low, especially in patients with normal heart rates or tachycardia, this finding suggests a decrease in vascular tone, and in these conditions, fluid administration alone is often insufficient to increase perfusion pressure.

In many patients, administration of vasopressors is thus required for this purpose.

Do vasopressors improve tissue perfusion? Increasing perfusion pressure is not equal to increasing tissue perfusion. First, once the autoregulatory threshold has been reached, further increasing perfusion pressure does not increase perfusion to the organ. Second, vasoconstricting agents may constrict arterioles, thereby decreasing microvascular perfusion. In healthy conditions, both norepinephrine and vasopressin decreased microvascular perfusion [6]. Accordingly, the net result of increasing perfusion pressure on tissue perfusion may depend on the balance between the potential beneficial effects on organ blood flow and negative impact on microvascular perfusion. Experimental evidence provides conflicting results, but factors such as the absence of fluid resuscitation or increased abdominal pressure may act as confounding factors. Evaluating the impact on tissue hypoperfusion in critically ill patients is not easy as it requires to perform advanced measurements before correction of hypotension. Introduction of norepinephrine in severely hypotensive septic shock patients is associated with an increase in cardiac output [7]. Several mechanisms may be implicated, including an increase in contractility, an increase in cardiac preload, and an increase in coronary artery perfusion [8]. The increase in blood pressure and cardiac output was associated with a decrease in lactate levels [7]. In a series of 14 patients with septic shock, correction of severe hypotension with norepinephrine administration resulted in an increase in mean arterial pressure (MAP) from 51 ± 3 mmHg to 79 ± 7 mmHg and was associated in the restoration of urine output in 12 of the 14 patients and an increase in creatinine clearance [9]. Even though evidence remains limited, these results suggest that norepinephrine improves tissue perfusion when used to correct severe hypotension. However, most of these trials evaluated the short-term effects of vasopressors and some of these beneficial effects may vanish over time. In addition, the optimal perfusion pressure may vary between patients.

## Optimizing cardiac output

**Fig. 2:**
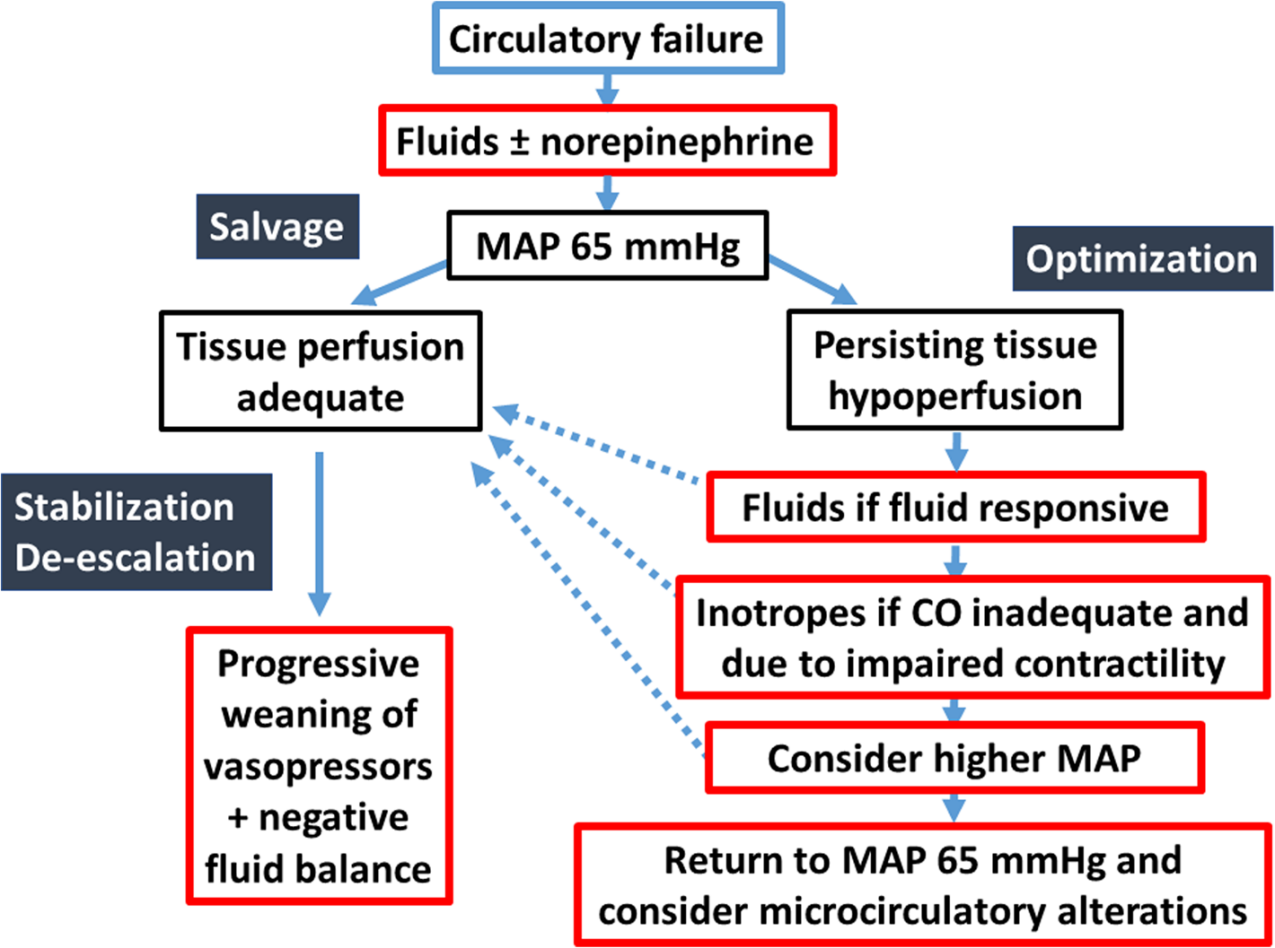
Suggested algorithm for tissue perfusion optimization. According to the phase of therapy (salvage/optimization/stabilization/de-escalation), different targets of resuscitation should be selected. Interventions should be progressively implemented, evaluating their effects and stopping it when ineffective. Once the patient is stabilized, weaning vasoactive agents and achieving a negative fluid balance should be considered

After the salvage phase aiming at restoring a minimal tissue perfusion pressure, optimizing cardiac output should be considered in order to improve tissue perfusion. Interestingly, cardiac output is not a target in itself. Indeed, it is very difficult to define what the ideal value of cardiac output is, as cardiac output fluctuates according to metabolic needs. Hence, instead of targeting given values of cardiac output, it is better to target adapted values of cardiac output. Cardiac output is judged inadequate based on ScvO_2_ and indices of tissue hypoperfusion. Increasing cardiac output requires a multiple steps approach (Fig. 2).

The first step should be to optimize preload in fluid responsive patients. Of note, all patients do not respond to fluids and excessive fluid administration is associated with a poor outcome [10]. Accordingly, it sounds appropriate to identify patients who may respond to fluids before fluid administration. The use of dynamic indices of fluid responsiveness, when applicable, allows the identification of fluid responders [11]. Do fluids also improve tissue perfusion? Obviously, the increase in cardiac output and tissue perfusion may be dissociated. Fluids improve microvascular perfusion only in the first 24 h, but not after 48 h, of sepsis recognition [12]. In a small series of patients, the first bolus improved cardiac output and microvascular perfusion while the second bolus failed to improve microvascular perfusion even though cardiac output further increased [13]. Hence, fluids improve microvascular perfusion in the early phases of sepsis and the optimal amount of fluids required to improve microvascular perfusion remains to be determined.

The second step is to consider inotropic agents when contractility is impaired and results into inadequate cardiac output and impaired tissue perfusion. Adrenergic inotropic agents are used to increase cardiac output aiming ultimately at restoring/improving tissue perfusion. While intuitive in its concept, this approach nevertheless implies several prerequisites. First, it implies that regional organ perfusion would increase in parallel to the increase in cardiac output. In patients with septic shock, increasing cardiac output with dobutamine was associated with an increase in hepato-splanchnic [14] or in cerebral perfusion [15]. The effects of dobutamine on other beds remain less studied.

Importantly, the use of inotropic agents requires prior optimization of cardiac preload.

The final step to consider is the optimization of cardiac afterload. While on the one hand one may consider to increase MAP in order to increase perfusion pressure, the increase in MAP also increases left ventricular afterload which may impair cardiac function and thus cardiac output, especially in patients with sepsis-associated myocardial depression [16].

## Which targets?

The targets of resuscitation include perfusion pressure and various indices of tissue hypoperfusion.

What is the optimal blood pressure target? Varpula et al. [2] first demonstrated in 111 patients with septic shock that the time spent with MAP below 65 mmHg was associated with a worse outcome. Several trials not only confirmed this finding [3, 17, 18], but even further expanded this reporting that MAP threshold levels lower than 65 were associated with even more severe outcomes. Interestingly, mortality already increases with shorter periods of time when thresholds of MAP lower than 65 mmHg are considered compared to the 65 mmHg threshold. On the contrary, there was no association between mortality and time spent with below MAP thresholds 80–85 mmHg (but above 65 mmHg) [3, 17, 18]. Altogether, these observational studies support a MAP threshold around 65 mmHg for the majority of the patients.

However, the optimal MAP threshold may require some individualization. In a series of patients with sepsis but without clinical signs of hypoperfusion, a MAP between 55 and 65 mmHg was well tolerated [19]. In an interventional trial evaluating a MAP threshold of 65–70 versus 80–85 mmHg, no differences in survival were observed. In the subgroup of patients previously hypertensive, the higher MAP target was associated with a reduction in acute kidney injury. However, there was also a significant increase in arrhythmic events and a trend toward an increase in acute myocardial infarction. This trial had two major limitations. First, MAP was much higher than the predefined target value in the lower threshold group so that the safety of MAP 65 was not really evaluated. Second, the trial attributed patients to one group or the other without considering the response to therapy. Interestingly, different MAP targets had variable effects on various indices of tissue hypoperfusion and organ function in several small size interventional studies [20, 21]. Hence, the recommendations of the Surviving Sepsis Campaign guidelines to initially target MAP of 65 mmHg remain valid [22]. In selected patients, a higher MAP may be considered, but the effects of increasing MAP above 65 mmHg should be carefully evaluated and returning to lower MAP thresholds should be considered if the patient does not respond to this MAP challenge.

Indices of tissue perfusion include cardiac output, SvO_2_ and ScvO_2_, veno-arterial differences in PCO_2_ (PvaCO_2_), skin mottling and capillary refill time, blood lactate levels, and urine output. While cardiac output is a major determinant of tissue perfusion, it is remarkable that no specific target value can be derived from the literature. However, it is important to ensure that interventions aiming at increasing cardiac output effectively increase it, to prevent the risk of excessive or even useless administration of fluids and inotropic agents.

Observational trials have shown that the time spent with SvO_2_ below 70% is associated with an increased risk of death. Trials targeting ScvO_2_ for resuscitation have been associated with variable outcomes [23, 24]. While the Rivers’ trial [23] showed some benefit with early goal-directed therapy based on ScvO_2_, these results were not confirmed in subsequent trials [24]. Of note, ScvO_2_ was already in the target at baseline in the latter trials so that the impact of goal-directed therapy could not be evaluated. On the other hand, the persistence of low ScvO_2_ is associated with a poor outcome [25]. Accordingly, even if the impact of goal-directed therapy can be questioned, the usefulness of ScvO_2_ to interpret hemodynamic data, and in particular adequacy of cardiac output, remains [26]. PvaCO_2_ can also be helpful in identifying patients with impaired tissue perfusion, especially when ScvO_2_ is close to the normal range [12, 27]. While PvaCO_2_ has a strong prognostic value [28], its use as a guide for resuscitation has not been tested in large-scale randomized studies. Nevertheless, it provides interesting additional information that should not be neglected. Clinical indices of tissue perfusion such as skin mottling and temperature or capillary refill time [29] also provide important information but, at this stage, have not been tested as a target to therapy. Lactate is an excellent marker of the balance between oxygen demand and actual oxygen consumption, the latter being affected by tissue perfusion. While increased blood lactate levels have consistently been associated with increased mortality rates [30], its hypoxic origin cannot be ascertained. Measuring repeatedly lactate and pyruvate, it has been shown that lactate is mostly of hypoxic origin in the first few hours after the onset of shock but that the proportion of patients presenting hyperlactatemia of hypoxic origin decreased over time so that the majority of the hyperlactatemia at 24 h are of non-hypoxic origin [31]. Hospital mortality significantly decreased in a trial targeting lactate decreases by 20% every 2 h for 8 h [32]. However, lactate decreases may be slow, especially in patients with impaired liver function. It sounds thus reasonable to target lactate decreases [22], but it should be coupled with other markers of tissue hypoperfusion [33]. Hence, many indices can be used to assess tissue hypoperfusion, but none seems superior to the other and it is difficult to have a predefined target value for most of these. Accordingly, it sounds wise to guide resuscitation aiming at improving multiple indices (i.e., ScvO_2_ + PvaCO_2_ + lactate + skin perfusion), acknowledging that some may fail to rapidly improve [33].

Even though promising, microcirculation assessment is not yet part of our clinical armamentarium. Several trials have shown that the microcirculation is altered, especially in sepsis, and that the severity of the alterations is associated with a poor outcome [34–36]. As alterations in microvascular perfusion cannot be predicted from systemic hemodynamics [37], it sounds interesting to evaluate the microcirculation. The main limitations to its current use are the lack of therapeutic intervention specifically targeting the microcirculation and the difficulty to apply these handheld microscopes on the sublingual area in certain conditions. Interestingly, PvaCO_2_ seems to be a good surrogate of microvascular perfusion [38] and may at least address one of these limitations.

Finally, different thresholds should be considered according to time. Globally, the care of the patient in shock can be divided into four phases: salvage, optimization, stabilization, and de-escalation [39]. The targets for therapy and interventions should be adapted according to these different phases (Fig. 2).

## Which agents?

For increasing perfusion pressure, administration of norepinephrine is associated with variable effects. Correcting severe hypotension with norepinephrine was associated with an improved organ function; however, increasing MAP from 65 to 75 or 85 mmHg was associated with variable effects. Second, and even more importantly, it implies that the catecholamine does not have negative effects on the microcirculation. While capillaries do not have adrenergic receptors, alpha- and beta-adrenergic receptors are present in resistive arterioles, at the entrance of capillary network. Administration of beta-adrenergic compounds is associated usually with an improvement in capillary perfusion. On the other hand, administration of alpha-adrenergic agents may be associated with a decrease in microvascular perfusion. The resulting effect of alpha-adrenergic administration on tissue perfusion will reflect the balance between increasing total organ blood flow and impairing microvascular perfusion.

Interestingly, epinephrine is associated with impaired splanchnic perfusion compared to norepinephrine [40]. In patients with cardiogenic shock, epinephrine was also associated with an impaired splanchnic perfusion. More importantly, epinephrine was associated with an increased incidence of refractory shock and a trend toward an increased mortality [41]. Even though the regional effects of norepinephrine and dopamine did not differ, dopamine is associated with more adverse effects and an increased risk of death [42, 43]. Accordingly, norepinephrine is considered as the first-line vasopressor agent [22].

Theoretically, it may be interesting to consider non-adrenergic vasopressors. Importantly, vasopressin derivatives as well as angiotensin behave similarly to norepinephrine. Indeed, even though these three agents stimulate different receptors at the surface of vascular smooth muscle cells, the downstream pathways are very similar with the implication of G protein, phospholipase C, and protein kinase C to promote increases in intracellular calcium through mobilization from sarcoplasmic reticulum and opening of voltage calcium channels. The difference between these agents is thus determined more by the density and sensitivity of the receptors than by the nature of the agent. At usual doses, the effects of norepinephrine and vasopressin do not differ; however, at high doses, vasopressin derivatives may be associated with digital necrosis [44] and splanchnic hypoperfusion [45]. Angiotensin administration may be associated with less requirements of renal support in patients with acute kidney injury [46], but these effects were noticed in a very small subgroup of patients and should be confirmed in larger trials.

Alternative approaches would be either to minimize exposure to vasopressors, tolerating hypotension and trying to prioritize perfusion but this approach can be valid as long as perfusion of the organs is preserved. Alternatively, one may consider to combine moderate doses of vasopressors, aiming at preserving organ perfusion, to vasodilatory agents in order to improve microvascular perfusion. In this context, nitroglycerin was shown to improve microvascular in eight patients with septic shock [47]. However, nitroglycerin was given together with a fluid bolus in order to minimize the risks of hypotension so that the effects of the fluid bolus in the improvement in microcirculatory perfusion cannot be excluded. A randomized trial failed to demonstrate any significant effects of nitroglycerin on the microcirculation in fluid-resuscitated septic shock patients. This may reflect the non-selective effect of nitroglycerin. More selective vasodilatory agents acting preferentially on non-perfused vessels have shown promising results in experimental conditions [48], but clinical evidence is still lacking.

For increasing cardiac output, dobutamine is the agent of choice. It is characterized by a short half-life and has minimal adverse effects at usual doses. In patients with septic shock, dobutamine increased oxygen delivery with minimal impact on MAP [49]. In most cases, the increase in cardiac output was related to an increase in stroke volume more. In another trial, it was nevertheless noted that the increase in cardiac output was in some case related more to an increase in heart rate than in stroke volume [50]. In addition, one should note that metabolic effects can be observed mostly when high doses of beta-adrenergic agents are used. Hence, one should always use the minimal dose associated with the desired hemodynamic effect and wean it as soon as possible. Non-adrenergic alternatives include phosphodiesterase inhibitors and levosimendan. Phosphodiesterase inhibitors like enoximone and milrinone are characterized by a long half-life and associate vasodilatory to the inotropic properties. Hypotension is often encountered and often limit/prevent the use of these agents in septic shock. Levosimendan, a calcium sensitizer, is an attractive alternative. Unfortunately, it is also associated with vasodilatory properties and with a very long half-life. This latter property makes this agent not optimal for therapy in septic patients as its action (positive inotropy as well as adverse effects) would remain present for 5–7 days. Of note, a prolonged effect is not desired in septic shock as myocardial depression usually resolves over a few days, and hence, indication for inotropic stimulation also disappears. In a small size trial, levosimendan was effective in increasing cardiac output and improving gastric perfusion [51]. Even though it has limited impact on blood pressure, the amount of fluids administered was also higher in patients receiving levosimendan. In a randomized trial, levosimendan was ineffective in preventing new-onset organ dysfunction [52]. Unfortunately, the indication to administrate levosimendan in that trial was not triggered on hemodynamic endpoints (no cardiac output measurements and no assessment of cardiac function). Indeed, as for other inotropic agents, levosimendan should only be administered in patients with tissue hypoperfusion related to an inadequate cardiac output associated with altered contractility. Accordingly, the hypothesis of whether levosimendan could improve, or not, tissue perfusion in patients with inadequate cardiac output in sepsis was not tested.

Dobutamine remains thus the inotropic agent of choice in sepsis, even though other inotropic agents may offer interesting alternatives in some conditions.

## Conclusions

The main goal of hemodynamic resuscitation in shock is to improve tissue perfusion and oxygenation. As these cannot be directly evaluated at bedside in routine practice, physicians are left with surrogates such as perfusion pressure, cardiac output, SvO_2_, ScVO_2_, PvaCO_2_, cardiac output, diuresis, lactate, and skin mottling. As none of these can be considered ideal and as no single target value can be identified for all patients, the management of the patients with septic shock patient should be individualized taking into consideration several indices of tissue hypoperfusion.

While catecholamines are often used to increase blood pressure and cardiac output, these have adverse effects that may be associated with increased risk of death especially when used at high doses. Accordingly, these agents should be wisely used, minimizing the dose by optimizing fluid resuscitation, considering alternative agents when possible, and taking into account the response to therapy.

## Abbreviations

MAP: Mean arterial pressure
PCO_2_: Partial pressure in CO_2_
PvaCO_2_: Veno-arterial difference in PCO_2_
ScvO_2_: Central venous oxygen saturation
SvO_2_: Mixed-venous oxygen saturation
